# *YULINK* regulates vascular formation in zebrafish and HUVECs

**DOI:** 10.1186/s40659-023-00415-8

**Published:** 2023-02-27

**Authors:** Hsin-Hung Lin, Ming-Wei Kuo, Tan-Chi Fan, Alice L. Yu, John Yu

**Affiliations:** 1 Chemical Biology and Molecular Biophysics Program, International Graduate Program, Academia Sinica, Taipei, Taiwan; 2 Institute of Stem Cell and Translational Cancer Research, Chang Gung Memorial Hospital at Linkou, 333 Taoyuan, Taiwan; 3 Department of Pediatrics, University of California, San Diego, CA USA; 4 Institute of Cellular and Organismic Biology, Academia Sinica, Taipei, Taiwan

**Keywords:** YULINK, Vasculogenesis, Endocytosis, Clathrin, VEGFR2, VEGF, RHOB, EPS15, RAB33B, TICAM2

## Abstract

**Background:**

The distinct arterial and venous cell fates are dictated by a combination of various genetic factors which form diverse types of blood vessels such as arteries, veins, and capillaries. We report here that YULINK protein is involved in vasculogenesis, especially venous formation.

**Methods:**

In this manuscript, we employed gene knockdown, yeast two-hybrid, FLIM-FRET, immunoprecipitation, and various imaging technologies to investigate the role of YULINK gene in zebrafish and human umbilical vein endothelial cells (HUVECs).

**Results:**

Knockdown of *YULINK* during the arterial-venous developmental stage of zebrafish embryos led to the defective venous formation and abnormal vascular plexus formation. Knockdown of *YULINK* in HUVECs impaired their ability to undergo cell migration and differentiation into a capillary-like tube formation. In addition, the phosphorylated EPHB4 was decreased in *YULINK* knockdown HUVECs. Yeast two-hybrid, FLIM-FRET, immunoprecipitation, as well as imaging technologies showed that YULINK colocalized with endosome related proteins (EPS15, RAB33B or TICAM2) and markers (Clathrin and RHOB). VEGF-induced VEGFR2 internalization was also compromised in *YULINK* knockdown HUVECs, demonstrating to the involvement of YULINK.

**Conclusion:**

This study suggests that YULINK regulates vasculogenesis, possibly through endocytosis in zebrafish and HUVECs.

**Supplementary Information:**

The online version contains supplementary material available at 10.1186/s40659-023-00415-8.

## Key points

1. Knockdown of *YULINK* with morpholino in embryos of double transgenic zebrafish exhibited abnormal venous formation.
2. Tube formation and phosphorylated EPHB4 were decreased in *YULINK* knockdown HUVECs.
3. FLIM-FRET, immunoprecipitation, as well as other imaging technologies showed that YULINK colocalized with endosome related proteins (EPS15, RAB33B and TICAM2) and endosome markers (Clathrin and RHOB).
4. Knockdown of *YULINK* decreased the internalization of VEGF and VEGFR2 in HUVECs.

**Supplementary Information:**

The online version contains supplementary material available at 10.1186/s40659-023-00415-8.

## Introduction

For the formation of blood vessels, the segregation of arterial and venous progenitors from a mixed, heterogeneous cell population is an important process for the establishment of arteries and veins [1, 2]. In the initial stages of vertebrate vascular development, there was a coordinated sorting and segregation of arterial and venous progenitor cells to become distinct networks of dorsal aorta and cardinal vein. The formation of the first axial artery and vein in the zebrafish embryo thus offers an excellent system to study the mechanism of vascular differentiation. By early embryonic development of zebrafish, endothelial progenitors initiate the expression of endothelial cell-specific genes and coalesce into a single vascular cord located at the position of the future dorsal aorta. Then, the lateral endothelial progenitors located in the dorsal aorta primordia sprout ventrally to form the first embryonic vein [3, 4]. The endothelial progenitors in which Notch and Erk signaling are activated are committed to a dorsal aorta fate [5, 6]. However, the molecular mechanism for venous vascular development is not yet known.

In our previous studies, zebrafish was used to study the functions of Ka/Ks-predicted novel human exons using comparative evolutionary genomics analysis [7–9]. From *in silico* analyses in the zebrafish, 308 potential genes that had no defined biological functions were found [10]. With a reverse genetic screening using genetic knockdown, we identified *YULINK* in zebrafish knockdown which displayed blood vessel abnormality. In addition, *YULINK* was reported in fly as “*mio”*, because it was required for the maintenance of the meiotic cycle [11]. It was also found in human HEK-293 T cells that YULINK is a subunit of GATOR2 complex proteins and inhibition of GATOR2 suppressed mTORC1 signaling [12]. More recently, YULINK was shown to be involved in cardiac function in zebrafish, mouse cardiomyocytes, and human iPSC-derived cardiomyocytes [13]. We showed that YULINK regulates Serca2 expression via PPARγ signaling and is involved in the development of human heart failure [13]. All studies suggest that *YULINK* is an evolutionarily conserved gene with diverse functions.

In this study, knockdown of YULINK gene led to defective venous formation in zebrafish embryos and impaired capillary tube formation in human umbilical vein endothelial cells (HUVECs). In addition, proteins interacting with YULINK were identified by yeast two-hybrid experiments and their colocalization in HUVECs was examined with FLIM-FRET (Förster Resonance Energy Transfer by Fluorescence Lifetime Imaging Microscopy) and other imaging methods. Treatment with endosome inhibitor led to abnormal venous formation in zebrafish and network formation in HUVEC cells. Furthermore, confocal or immunofluorescent microscopy analysis of HUVECs showed that *YULINK* knockdown affected the internalization of VEGF and VEGFR2, suggesting the involvement of YULINK in endocytosis.

## Results

### Knockdown of YULINK gene led to defective venous formation in zebrafish 

**Fig. 1:**
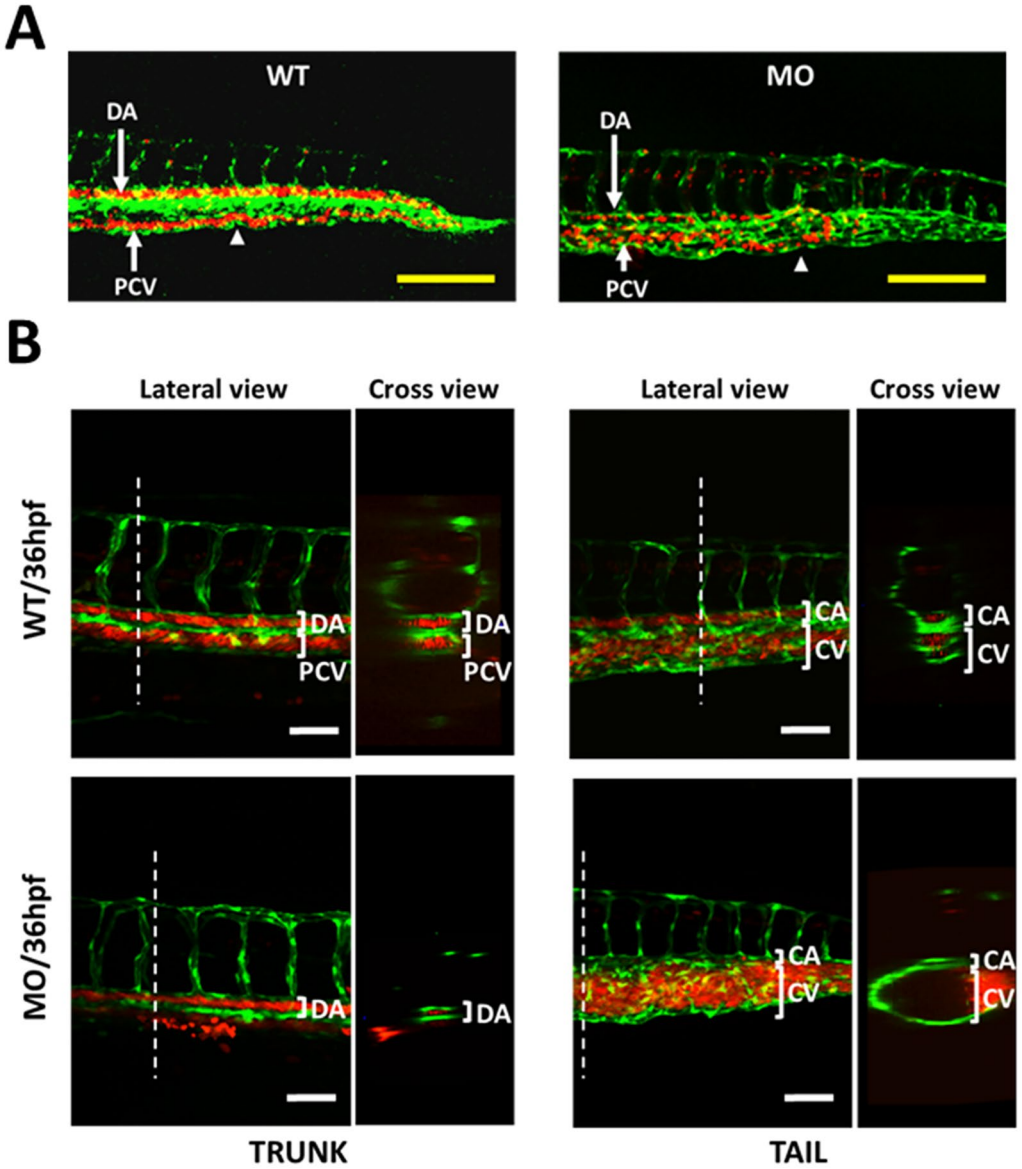
Effects of *YULINK* knockdown on the vasculature in zebrafish embryos. ** A** The double transgenic zebrafish *Tg (fli1:EGFP; gata1:DsRed)* displayed *fli1* promoter-derived green fluorescent for endothelial cells and *gata1* promoter-derived red fluorescent for erythrocytes. Arrows indicate the anus of the embryo. Yellow scale bars indicate 200 μm. **B** The confocal images of the trunk and caudal vasculature at 36 hpf are shown with their lateral and cross-section views. The white dotted lines indicate the position where the cross-sections were taken. *DA* dorsal aorta, *PCV* posterior cardinal vein, *CA* caudal artery, *CV* caudal vein. White scale bars indicate 50 μm

To investigate the role of YULINK on vascular development, two zebrafish transgenic lines, *Tg (gata1:DsRed)* and *Tg (fli1:EGFP)*^*y1*^, were crossed to generate double transgenic zebrafish *Tg (fli1:EGFP; gata1:DsRed)*. In Fig. 1A, this double transgenic zebrafish displayed endothelial cells with *fli1* promoter-derived green fluorescent and erythrocytes with *gata1* promoter-derived red fluorescent, thus enabling the analysis of the development for vasculature and blood circulation. As shown in Additional file 2: Video S1, wild type zebrafish embryos at 24 hpf showed that blood circulated through dorsal aorta (DA) to the tail end and flowed back into the posterior cardinal vein (PCV).To examine the biological function of the YULINK gene, zebrafish embryos were injected with antisense oligonucleotides morpholino (MO) to knockdown (KD) the expression of *YULINK*. It was found that blood in the *YULINK*-KD morphants did not ran all the way to the tail end and turned around earlier to reach PCV (Fig. 1 A and Additional file 2: Video S1); in more severely affected *YULINK*-KD morphants, there appeared to have complete loss of blood flow.

The lateral and cross-section views of the truck portion of the wild-type embryos indicated that DA and PCV were fully developed at 36 hpf with blood flow (Fig. 1B). By comparison, in the severely affected *YULINK*-KD morphants, only DA was shown; but PCV were not observed (Fig. 1B). On the other hand, in the tail portion of wild type zebrafish embryos, there observed one caudal artery (CA) and 2–3 caudal veins (CV), which constituted the caudal vein plexus with a complex venous vascular network (Fig. 1B). In the tail portion of the *YULINK*-KD morphants with normal CA, there was obvious accumulation of erythrocytes (red), and a large single caudal vein observed (Fig. 1B).

Whole-mount in situ hybridization employing arterial *efnb2a* and venous *dab2* markers was used to distinguish arterial and venous vasculature in zebrafish [14, 15]. The lateral view of wild type embryos and *YULINK*-KD morphants both displayed the arterial endothelial marker, *efnb2a* (Additional file 1: Fig. S1A). Compared to the expression of venous marker, *dab2*, in the wild type embryos at 2 dpf, there was no expression of the venous marker in the trunk region of *YULINK*-KD morphants (Additional file 1: Fig. S1A), suggesting that PCV did not develop normally in the truck region and consistent with the lack of PCV expression in *YULINK-*KD morphants in this region.

The arterial and venous vascular networks in the trunk region were further analyzed using another double transgenic zebrafish. In this study, two transgenic lines, *Tg (fli1:EGFP)*^*y1*^ in which endothelial cells were labelled with *fli1* promoter-derived green fluorescent, and *Tg (flt1*^*enh*^:*RFP)* line, in which the arterial endothelial cells were labeled with *flt1* promoter-derived red fluorescence, were crossed and generated the other double transgenic zebrafish. Such generated double transgenic embryos displayed yellow color for arteries (in combination of red arterial endothelial cells and the green endothelial cells) and green color for veins (Additional file 1: Fig. S1B). Therefore, in the wide type embryos at 2 dpf, the DA and the arterial intersegmental vessels (aISV) were shown with yellow color, whereas the PCV and venous intersegmental vessels (vISV) exhibited green color (Additional file 1: Fig. S1B). In contrast, in the morphants the large DA and thin aISV, which had either red or yellow color, were observed, suggesting the arterial development; but no green PCV and vISV were observed in these morphants. These studies thus suggested that knockdown of *YULINK* led to the loss of venous vasculature.

### Effects of *YULINK* knockdown in HUVECs

**Fig. 2:**
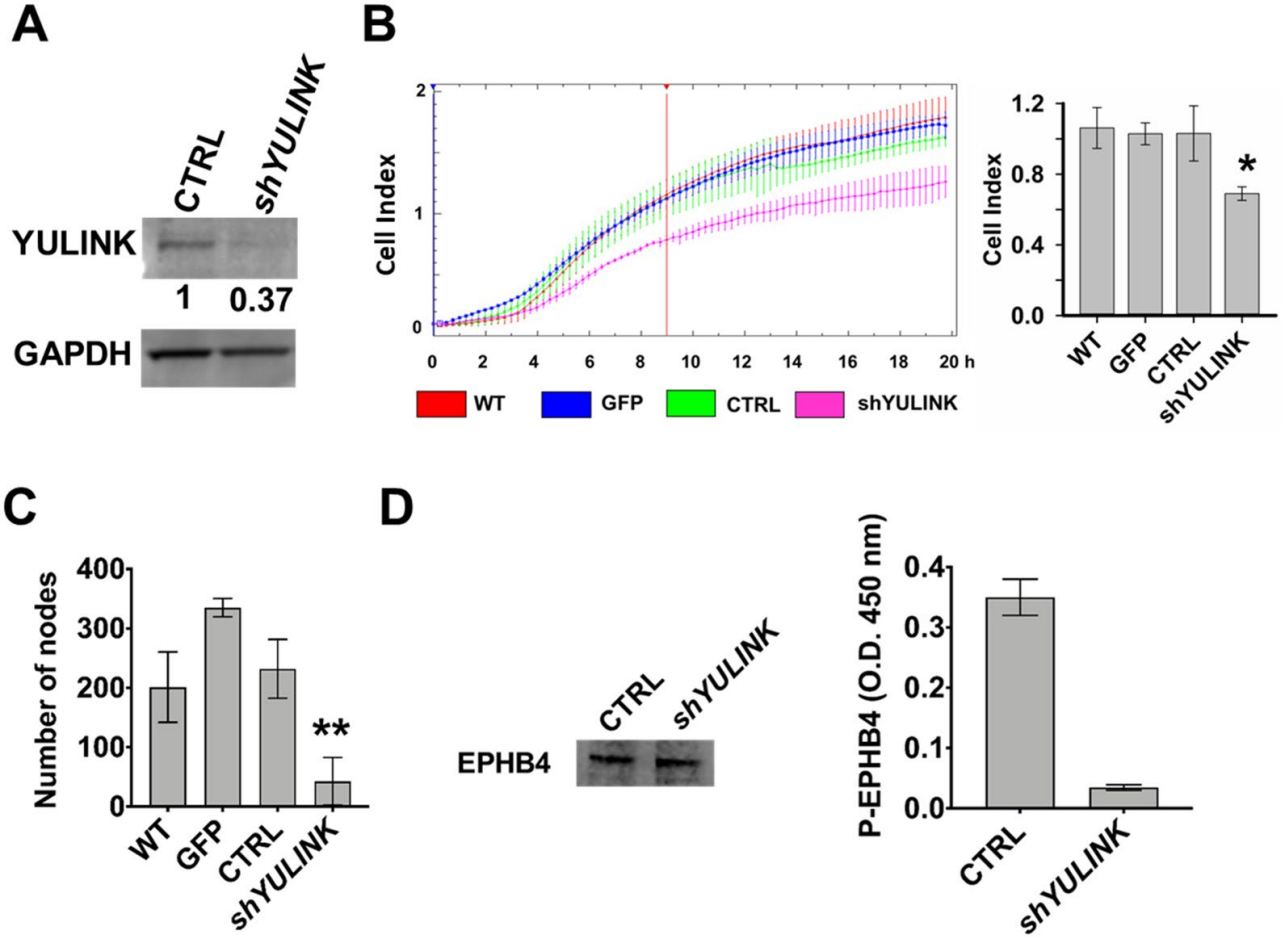
Effects of *YULINK*-knockdown on formation of capillary-like tubes, migration, and level of phosphorylated EPHB4 in HUVECs. HUVECs were transfected with plasmids containing GFP, vector-control (CTRL), or *YULINK* shRNA (*shYULINK*) for 1 day.** A** The knockdown efficiency of *YULINK* shRNA was analyzed at 1 day after transfection in Western blot using anti- YULINK antibody. GAPDH was used as a loading control. The intensity of the YULINK band was quantified and normalized to the GAPDH.** B** Real-time cell migration of the transfected cells was measured using CIM plates in the xCELLigence DP system, which detects the impedance across a cell-permeable membrane. HUVECs were transfected with plasmids containing GFP, vector-control (CTRL), or *YULINK* shRNA (*shYULINK*). All cells were seeded (1.6 × 10^4^ cells/well) and allowed to migrate for 20 h (left panel). Cell migration activity, expressed as cell index, was determined 9 h later (right panel, mean ± SD, *n* = 3, *p < 0.05). WT, wild type untreated cells.** C** Number of nodes were measured in transfected cells determined using a Matrigel-embedded tube forming assay (mean ± SD, *n* = 3, **p < 0.001).** D** Expression of the venous marker EPHB4 in total cell lysates was examined by Western blot and served as the internal control (left panel). Expression of phosphorylation EPHB4 was examined by ELISA (right panel). The ELISA optical density (O.D.) was determined at the visible wavelength 450 nm (mean ± SD, *n* = 6, **p < 0.001)

The effects of *YULINK*-knockdown on endothelial cell migration and network formation of branching and capillary-like tubes were then examined. HUVECs were transfected with plasmids containing GFP, vector-control (CTRL), or *YULINK* shRNA (*shYULINK*) for 1 day. Western blot confirmed efficient suppression of YULINK by shRNA, and the protein level of YULINK remained 37% in *YULINK*-knockdown HUVECs (Fig. 2 A). In addition, quantitative-PCR also confirmed specific suppression of *YULINK* by the shRNA, and the mRNA level of YULINK remained 39% in *YULINK*-knockdown HUVECs (Additional file 1: Fig. S2A).

Cell migration was real-time monitored using CIM-Plate 16 devices and the xCELLigence DP system [16]. In this system, HUVECs were transfected with plasmids containing GFP, vector-control (CTRL), or *YULINK* shRNA (*shYULINK*) and monitored over a period of up to 20 h, by measuring changes in the impedance signal of the underside of the CIM-plate membrane. The quantitative comparison of the migration assayed at 9 h was expressed as cell index (Fig. 2B) among various groups and shown in bar graph on the right (*n* = 3, mean ± SD). The p-value between shYULINK and CTRL was less than 0.05 (*) and p-values among WT, GFP and CTRL were not significantly different. The knockdown of *YULINK* significantly decreased migratory capacity about 33.0%, as shown in Fig. 2B. Network formation was assessed after 16 h by photographing the matrices using an inverted light microscope and digital camera. In addition, knockdown of *YULINK* significantly inhibited formation of capillary-like tubes, as measured the number of nodes and determined using a Matrigel-embedded tube forming assay (Fig. 2C and Additional file 1: Fig. S2B–F). A reduction in the basal levels of *YULINK* led to decreased network formation as judged by number of nodes in Matrigel about 81.6% compared with control (Fig. 2 C). Furthermore, network formation, as judged by total length, number of junctions and total branching length, was significantly lower about 42.8%, 81.8% and 71.7% compared with control, respectively (Additional file 1: Fig. S2B–F). These experiments thus suggest that YULINK regulates cell migration and network formation of branching and capillary-like tubes in HUVECs.

### Effects of ***YULINK***-knockdown in EPHB4 signaling

The B4 ephrin receptor (EPHB4) has been identified as a marker for veins [17]. To determine whether YULINK influenced EPHB4 signaling, we examined the expression and phosphorylation of the venous marker EPHB4 in *YULINK*-knockdown HUVECs. The HUVECs were treated with shRNA (*shYULINK* or vector control (CTRL)), then the expression and phosphorylation of the venous marker EPHB4 in total cell lysates were examined by Western blot and ELISA. It was found that phosphorylated EPHB4 was decreased in *YULINK*-knockdown cells compared to the control cells, although the EPHB4 expression level remained the same as the control cells (Fig. 2D). Therefore, these studies demonstrate the key role of YULINK in vasculogenesis.

### YULINK interacting proteins by yeast two-hybrid assay and FLIM-FRET analysis

The yeast two-hybrid assay was used to identify proteins interacting with YULINK in yeast system. The yeast two-hybrid library was obtained from commercial human cDNA library, in which abundant cDNAs with high-copy-number were removed to facilitate the identification of novel protein-protein interactions. After yeast two-hybrid screening, blue colonies that had been selected were used for PCR and sequencing. There are six YULINK interacting proteins identified as EPS15, RAB33B, TICAM2, ANKRD44, DENND4C and LCA5L (Additional file 1: Table S1). Among these candidates, EPS15, RAB33B, and TICAM2 were chosen for further characterization, because these proteins were implied to be involved in endocytosis based on previous studies [18–22].

To confirm the interactions of YULINK with these three proteins in live cells, YULINK was conjugated with AcGFP (AcGFP-YULINK), and the putative interacting proteins, EPS15, RAB33B, and TICAM2, were conjugated with DsRed. Then, they were co-expressed in HEK-293 T, and the FRET (Förster Resonance Energy Transfer) of AcGFP was measured using multi-photon fluorescence lifetime imaging microscopy (FLIM). While transferring energy from an excited donor (AcGFP) to an acceptor (DsRed), FRET decreases the donor fluorescence and increases the acceptor fluorescence. The energy transfer between the donor and acceptor fluorophores can occur only when the two proteins are very close together (~ 1 to 10 nm), whereby the level of FRET Efficiency (shown as E) is proportional to the distance of interacting proteins [23]. Due to this proximity dependence, the most quantitative readout of a FRET interaction is provided by measuring the “mean fluorescence lifetime (τ)” of the donor fluorophore, defined as the mean time between fluorophore excitation and photon emission, which is called FLIM [24].

**Fig. 3:**
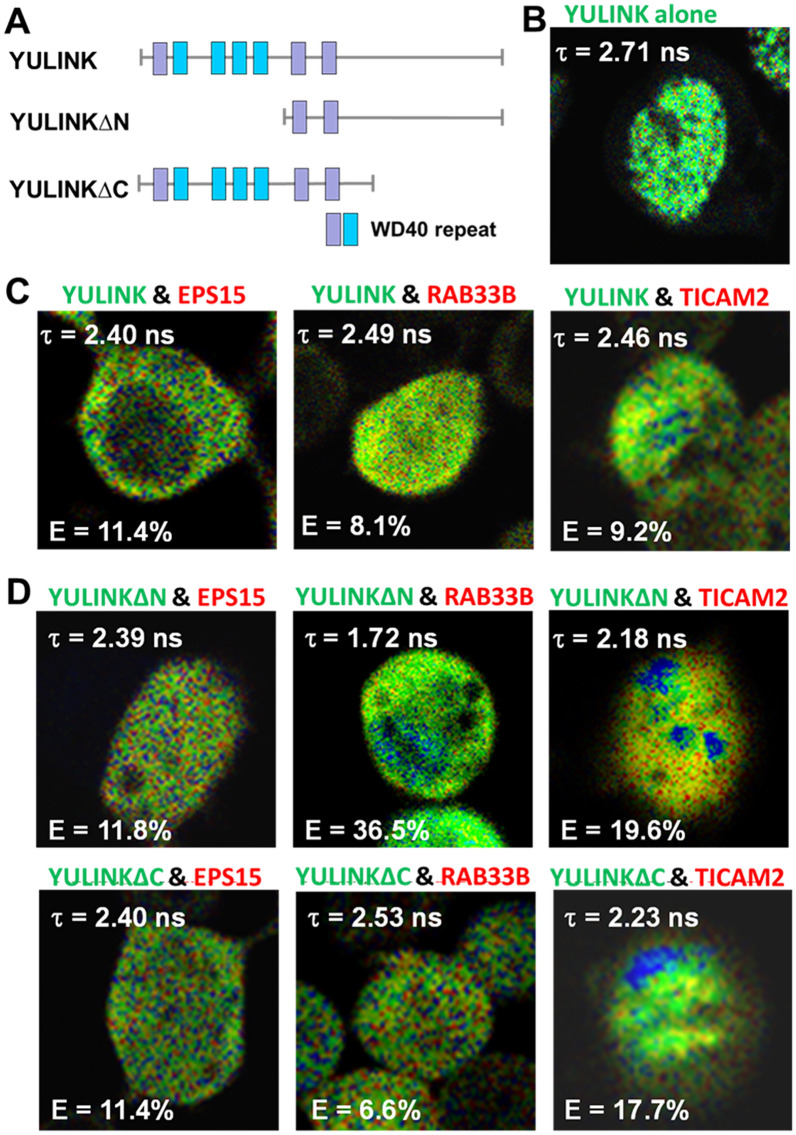
FLIM-FRET analysis of proteins interacting with YULINK.** A** Plasmid construct for full-length YULINK protein containing four conserved WD40 repeats (blue) and 3 potential WD40 candidates (purple). Other plasmid constructs for truncated forms of N- or C-termini (YULINK∆N or YULINK∆C) are also shown.** B**–**D** To validate these interactions, YULINK (full-length or truncated forms) was conjugated with AcGFP and the interacting protein candidates, EPS15, RAB33B, or TICAM2, were conjugated with DsRed, separately. Then, they were co-expressed in HEK-293 T cells and the FRET (Förster Resonance Energy Transfer) of AcGFP was measured using multi-photon fluorescence lifetime imaging microscopy (FLIM). The mean fluorescence lifetime (τ) and the FRET efficiency E (%) were measured at 48 h after co-transfection of HEK-293 T cells with AcGFP-YULINK (full-length or truncated forms) and DsRed-EPS15, DsRed-RAB33B, or DsRed-TICAM2, respectively. The less mean fluorescence lifetime (τ) and more FRET efficiency E (%) indicated stronger interaction and shorter distance between AcGFP and DsRed. The fluorescence lifetime (τ) of cells expressing only AcGFP-YULINK was as a FLIM-FRET control

Previous study[13] showed that YULINK protein possessed four conserved WD40 repeats (blue) and three putative WD40 repeats (purple) (Fig. 3 A). In addition, constructs of the N-terminus truncated form, YULINKΔN and the C-terminus truncated form, YULINKΔC, were shown in Fig. 3 A. The FRET between full-length AcGFP-YULINK and various DsRed conjugates (DsRed-EPS15, DsRed-RAB33B, or DsRed-TICAM2) were shown in Fig. 3B-D. The τ and the FRET efficiency (E), calculated by comparing the FLIM values obtained for the AcGFP donor fluorophore in the presence and absence of the DsRed acceptor fluorophore, reflected the interaction and the distance between AcGFP and DsRed. The τ of the cells expressing AcGFP-YULINK was approximately 2.71 ns (Fig. 3B), similar to that for AcGFP alone. Co-expression of AcGFP-YULINK and DsRed-EPS15 in HEK-293 T cells changed the τ to be 2.40 ns (E = 11.4%, Fig. 3 C), suggesting the mutual interaction between YULINK and EPS15. Similarly, co-expression of AcGFP-YULINK and DsRed-RAB33B or DsRed-TICAM2 also shift in τ to be 2.49 ns (E = 8.1%) and 2.46 ns (E = 9.2%), respectively. These results support the notion that EPS15, RAB33B, and TICAM2 interacted with YULINK in human cells.

Co-expression of YULINKΔN with DsRed-ESP15, DsRed-RAB33B, or DsRed-TICAM2 resulted in a decrease of τ to be 2.39 ns (E = 11.8%), 1.72 ns (E = 36.5%), or 2.18 ns (E = 19.6%) (Fig. 3D). Similarly, co-expression of YULINKΔC with these DsRed-conjugates, also caused a decrease of τ to 2.40 ns (E = 11.4%), 2.53 ns (E = 6.6%), or 2.23 ns (E = 17.7%) (Fig. 3D). Compared to the full-length YULINK, the FRET efficiency (E) between YULINKΔN and RAB33B was increased from 8.1 to 36.5%, and the FRET efficiency between YULINKΔN and TICAM2 was increased from 9.2 to 19.6%, showing that the N-terminus truncated YULINK was closer to the interacting protein RAB33B and TICAM2, respectively (E is increasing when two proteins are closer). For the C-terminus truncated YULINK, the FRET efficiency (E) between YULINKΔC and RAB33B was decreased from 8.1 to 6.6%, and the FRET efficiency between YULINKΔC and TICAM2 was increased from 9.2 to 17.7%, showing that the C-terminus truncated YULINK was only closer to the interacting protein TICAM2. Regarding the N-terminus truncated form or the C-terminus truncated form of YULINK interacted with EPS15, the truncated region of YULINK needs to be optimized. Based on these findings, it was suggested that these proteins, EPS15, RAB33B and TICAM2 were interacting with YULINK in cells.

#### Immunoprecipitation studies for YULINK-interacting proteins

Alternatively, co-immunoprecipitation was used to further confirm that these putative-interacting proteins form specific complexes with YULINK. First, YULINK was tagged with AcGFP and over-expressed in HUVECs by transfection. Then cell lysates were immunoprecipitation with anti-GFP antibody (Additional file 1: Fig. S3). Immunoblot analysis showed that exogenously expressed YULINK co-purified with the endogenous YULINK-interacting proteins EPS15, RAB33B, or TICAM2. Altogether, these results suggest that YULINK interacts with EPS15, RAB33B, or TICAM2 *in vivo.*

#### Super-resolution microscopic and confocal microscopy analysis with endocytosis related proteins

**Fig. 4:**
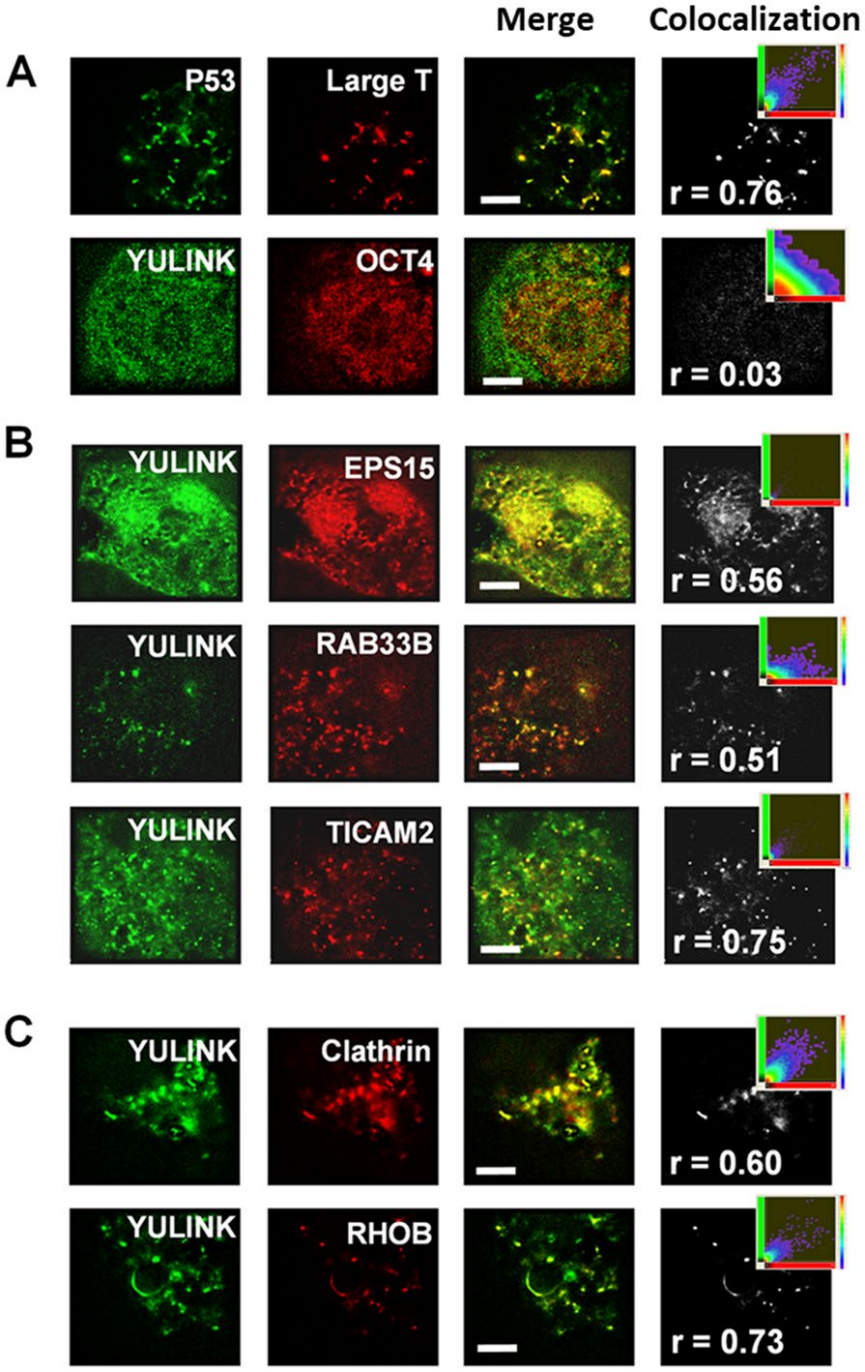
Analysis of colocalization using super-resolution microscopy. **A**, **B** The HEK-293 T cells co-expressing AcGFP-YULINK (green) and DsRed conjugates of the proteins of interest (red) were analyzed for colocalization ratio using super-resolution microscopy at 2 days after co-transfection. Merge images (yellow) show colocalization of AcGFP (green) and DsRed (red). Projection images of the colocalization (gray) were from merge images and shown in the far-right column of each row. The inset scatterplots indicated the extent of colocalization within projection images. Higher Pearson correlation coefficient (r) indicated more colocalization ratio. **C** Colocalization of YULINK and endosome markers (Clathrin or RHOB). The HEK-293 T cells co-expressing AcGFP-YULINK and mKate-Clathrin, or AcGFP-YULINK and DsRed-RHOB, respectively. Scale bars indicate 4 μm

Super-resolution and confocal microscopy were used to visualize whether the identified proteins physically colocalized with YULINK using Leica SR-GSD and TCS-SP5-MP-SMD confocal microscopes (Fig. 4 and S4A). As shown in FLIM-FRET analysis, YULINK was conjugated with AcGFP (AcGFP-YULINK), and the putative interacting proteins, EPS15, RAB33B, and TICAM2, were conjugated with DsRed. The HEK-293 T cells, was co-transfected with various plasmids carrying AcGFP- or DsRed-conjugated fluorescence, as described in details below.

In a Leica SR-GSD microscope, images were taken at a rate of 100 frames per second and reconstructed from a series of ~ 5,100 images, giving a total measurement time of about 1 min for each color channel. Quantitative colocalization ratio analysis of multicolor fluorescence images was performed with the Imaris software. In the super-resolution microscopic analysis, the Pearson correlation coefficient (r) indicated colocalization ratio. The binding of P53 (AcGFP-P53) with simian virus 40 large T antigen (DsRed-large T) in HEK-293 T cells was used as a positive control [25], which showed a high r = 0.76 (Fig. 4A). On the other hand, since there was no interaction between YULINK and OCT4 (as a negative control), the Pearson correlation coefficient was about 0.03 (Fig. 4A).

Then the HEK-293 T cells were transfected with co-expressing of YULINK and the DsRed conjugates of the EPS15, RAB33B, or TICAM2 and analyzed using super-resolution microscope. In Fig. 4B, the Pearson correlation coefficient r values for the AcGFP-YULINK and the DsRed-EPS15, DsRed-RAB33B, and DsRed-TICAM2 pairs, was respectively, 0.56, 0.51, and 0.75. These results strongly suggest that YULINK was colocalized with EPS15, RAB33B, or TICAM2.

In addition, HUVECs were fixed, permeabilized, stained with antibodies against the proteins of interest and analyzed by confocal microscopy. The colocalization rates showing that the signal ratio from both channels (green and red) overlap each other. The colocalization rates were 43.2%, 89.6%, and 58.9% between YULINK and EPS15, RAB33B, or TICAM2, respectively (Additional file 1: Fig. S4A). Altogether these image analyses confirmed the colocalization of YULINK with EPS15, RAB33B, or TICAM2.

Previous work has shown that EPS15 plays a role in endocytosis, and that RAB33B and TICAM2 may also be involved [18]. It is thus suggested that YULINK may be involved in endocytosis regulation. To test this hypothesis, we examined whether YULINK is also colocalized with endosome markers, Clathrin and RHOB [18, 26]. As shown in Fig. 4 C, the HEK-293 T cells were co-transfected with AcGFP-YULINK and mKate-Clathrin, or AcGFP-YULINK and DsRed-RHOB, and carried out on a Leica SR-GSD super-resolution microscope, respectively. The colocalization ratio between YULINK and Clathrin, or YULINK and RHOB pairs were revealed by the projection images of the colocalization showing gray color, and the colocalization correlation coefficient values of 0.60 or 0.73, respectively (Fig. 4 C). In addition, the association of AcGFP-YULINK and mKate-Clathrin was also examined using confocal microscope, which exhibit yellow staining, suggesting the association of Clathrin and YULINK (71.3% with r = 0.83 in Additional file 1: Fig. S4A).

Then, we sought to verify the subcellular localization of Clathrin and the identified YULINK-interacting proteins (EPS15, RAB33B, and TICAM2) in HUVECs with immunostaining. As shown in Additional file 1: Fig. S4B, staining with specific antibodies revealed colocalization of Clathrin with all three proteins and the colocalization rates for Clathrin/EPS15, Clathrin/RAB33B, and Clathrin/TICAM2 were 86.6%, 95.7% and 87.3%, respectively. These experiments thus suggest that YULINK and the YULINK-interacting proteins, EPS15, RAB33B, and TICAM2 may all be involved in endocytosis.

Next, we used *YULINK*-knockdown HUVECs to do rescue experiments with over-expression of DsRed-EPS15, DsRed-RAB33B or DsRed-TICAM2. Real-time cell migration of the transfected cells was measured using CIM plates in the xCELLigence DP system. The quantitative comparison of the migration assayed at 9 h was expressed as cell index. The p-value was less than 0.01 (**) between shYULINK and shYULINK + EPS15 and p-value was less than 0.05 (*) between shYULINK and shYULINK + RAB33B (Additional file 1: Fig. S5). However, over-expression of another endosome related protein, TICAM2, in *YULINK*-knockdown HUVECs did not show statistically significant difference in migration. These data thus suggested that over-expression of some endosome related proteins can rescue phenotype of *YULINK*-knockdown cells.

#### Effects of endocytosis inhibitor in zebrafish

**Fig. 5:**
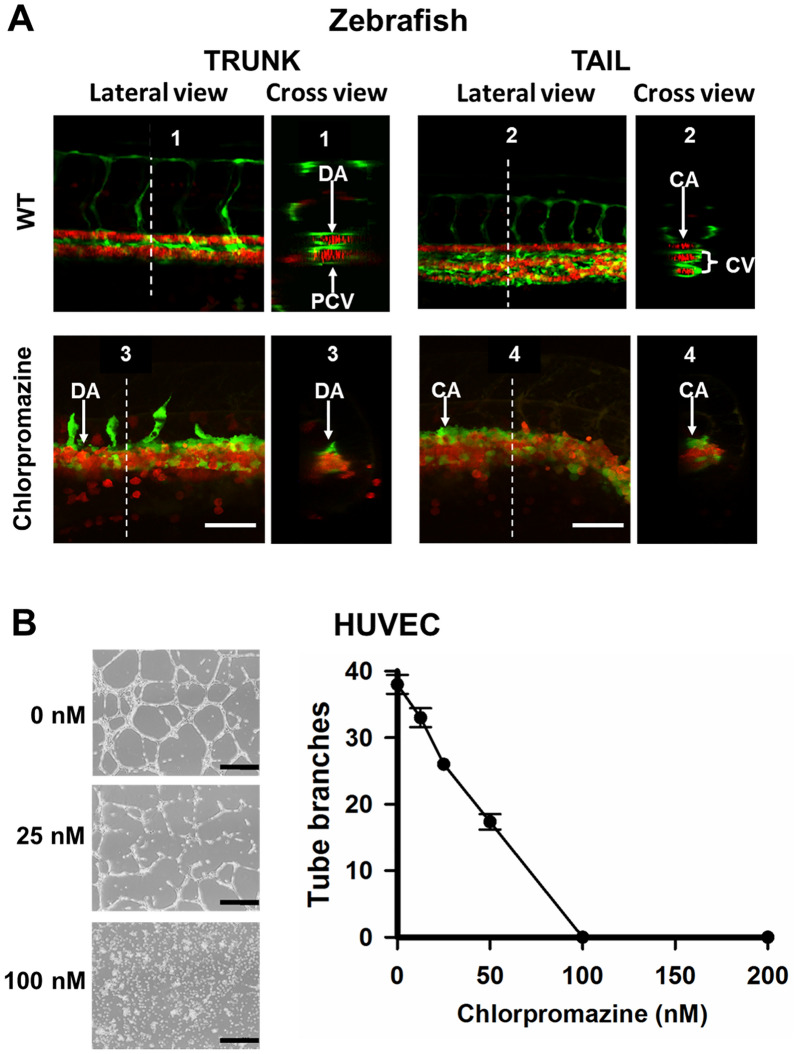
Effects of an endocytosis inhibitor on vein morphogenesis in vivo and capillary tube formation in vitro. ** A** The double *Tg (fli1:EGFP; gata1:DsRed)* embryos were treated with endocytosis inhibitor, chlorpromazine (60 nM), at 16 hpf. Chlorpromazine was removed at 22 hpf. The lateral and cross-section views of blood vessels in trunk and tail of the *Tg (fli1:EGFP; gata1:DsRed)* embryos at 24 hpf were shown. White dotted lines indicate the position where the cross-sections are taken. The upper panels were shown as WT groups. *DA* dorsal aorta, *PCV* posterior cardinal vein, *CA* caudal artery, *CV* caudal vein. Scale bars indicate 50 μm. **B** Chlorpromazine-mediated inhibition of capillary tube formation in vitro. HUVECs were pretreated with different concentrations of chlorpromazine for 1 h and then seeded in 12-well plates coated with Matrigel. Representative areas are photographed at 10× magnification. Black scale bars indicate 400 μm. Data points represent mean ± SD of two replicate experiments with 4 measurements per treatment

To further determine whether suppression of endocytosis affects venous formation, the effects of endocytosis inhibitor on vessel formation in zebrafish and on tube formation of HUVEC cell were examined. Chlorpromazine was known to inhibit clathrin-coated pit-mediated endocytosis by preventing the assembly of clathrin adaptor protein-2 (AP-2) [27]. For the following experiment, the double transgenic embryos (*fli1:EGFP; gata1:DsRed*) were treated with chlorpromazine (60 nM) at 16 hpf. Then, the inhibitor was removed at 22 hpf. In zebrafish embryos, the trunk was the part of the body posterior to the head and anterior to the tail region, which include dorsal aorta and posterior cardinal vein. The tail was the part of the body posterior to the anus which includes the anal and caudal fins. The lateral and cross-section views of the truck and tail portions of the wild-type embryos showed that DA, PCV, CA and CV were fully developed at 24 hpf (Fig. 5 A, upper two panels). It was found that chlorpromazine treatment for the embryos resulted in completely defective formation of PCV in the trunk region and CV in the tail region (Fig. 5 A, lower two panels). These results showed that inhibition of endocytosis interfered with arterial-venous formation, leading to abnormal vascular plexus formation.

To investigate the role of endocytosis in network formation, HUVECs were pre-treated with different concentrations of chlorpromazine. As shown in Fig. 5B, chlorpromazine dramatically suppressed capillary-like network formation in a dose-dependent manner, with complete disruption of the capillary network at 100 nM. Thus, endocytosis is required for tube formation of endothelial cells in vitro and formation of veins in vivo.

#### Involvement of VEGF signaling pathway in HUVECs

**Fig. 6:**
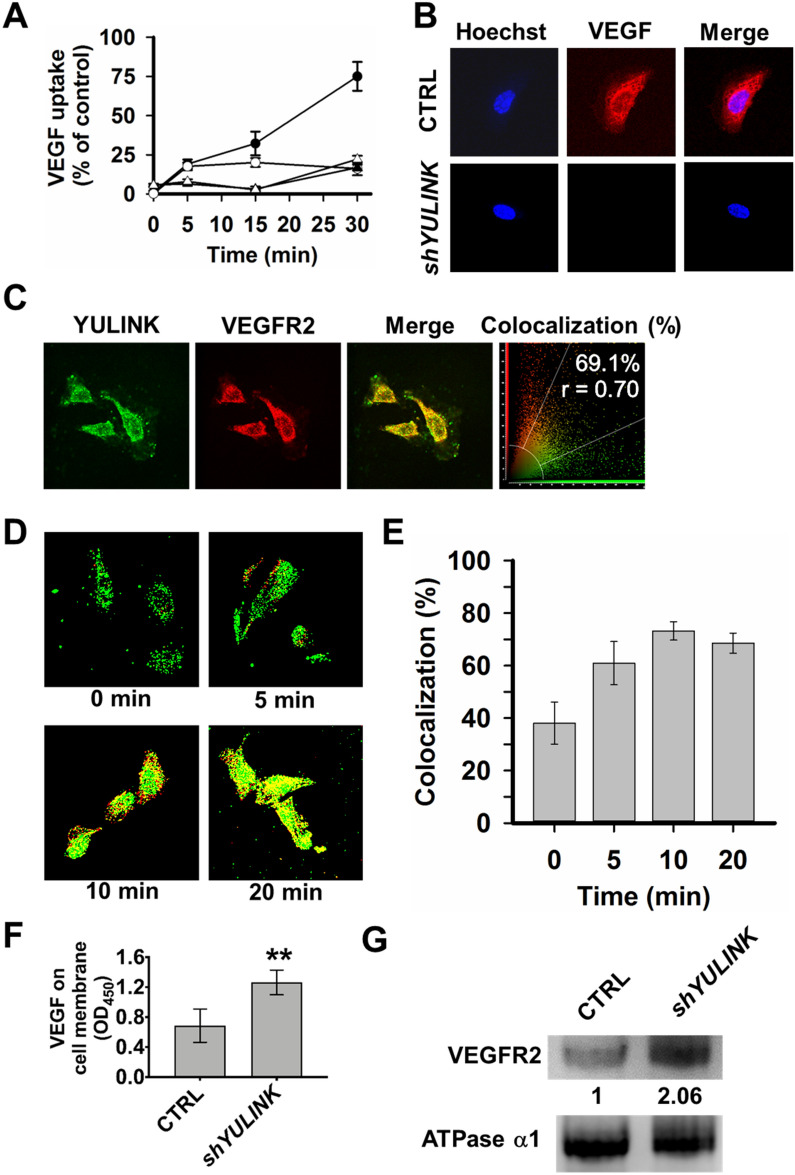
YULINK mediates VEGF internalization and VEGFR2 trafficking in HUVECs. **A**,** B** CTRL- and *YULINK*-knockdown HUVECs were incubated with VEGF-biotin and anti-VEGF blocking antibody pretreated with VEGF-biotin for the indicated times (5, 15, and 30 min). Cells were then fixed and stained with Alexa-Fluor-594-conjugated streptavidin for analysis by confocal microscopy. **A** Quantified VEGF intensity in cells at various time points. The average intensity of the VEGF signal per cell was calculated from at least twenty cells for each group. ●, CTRL. ○, *shYULINK*. ▲, CTRL with anti-VEGF blocking antibody. △, *shYULINK* with anti-VEGF blocking antibody. **B** Confocal fluorescence images of internalized VEGF in vector control (upper row) and *YULINK*-knockdown cells (lower row) at 30 min. Hoechst, cell nucleus staining. **C** HUVECs were stained for YULINK (green) or VEGFR2 (red). Overlay images demonstrate colocalization of green and red-stained molecules by a shift towards yellow in a Leica confocal system. The percentage of colocalizing pixels was shown in the far right column. Colocalization rate and Pearson correlation coefficient (r) were determined for pixel intensity correlation between the red and green channels. The data reveal a high percentage of colocalizing pixels for YULINK/VEGFR2. **D** VEGFR2 internalization was characterized in serum-starved HUVECs with VEGF treatment for the indicated times, before being fixed, permeabilized, labeled with anti-YULINK (green) and anti-VEGFR2 (red) antibodies, and visualized using fluorescent microscopy. **E** Quantification of VEGFR2/YULINK colocalization ratios were analyzed at each time point from panel D (mean ± SD). Data points represent mean ± SD of two replicate experiments with 25 measurements per timepoint. **F** HUVECs treated with shRNA against *YULINK* or vector control (CTRL) were incubated with VEGF-biotin for 30 min. Then HRP-conjugated streptavidin and TMB were added and OD_450_ value was measured.** G** The amount of VEGFR2 at the membrane fraction in the lysate of HUVECs which were treated with shRNA against *YULINK* or vector control (CTRL) was shown in western blot. Na^+^/K^+^ ATPase a1 was used as an internal control

To address whether YULINK is involved in VEGF/VEGFR signaling, we examined whether knockdown of *YULINK* affected the internalization of VEGFR2 when VEGF signaling was stimulated in cultured endothelial HUVECs (Fig. 6). The CTRL and *YULINK*-knockdown HUVECs were incubated with VEGF-biotin and anti-VEGF blocking antibody pretreated with VEGF-biotin. Then, these cells fixed and stained with Alexa-Fluor-594-conjugated streptavidin for analysis by confocal microscopy or fluorescent microscopy. The uptake of VEGF was observed in most CTRL cells, and decreased uptake of VEGF was shown in *YULINK*-knockdown cells from 0 to 30 min (Fig. 6 A and B). Moreover, we also found that YULINK is colocalized with VEGFR2 in HUVECs (69.1% with r = 0.7 in Fig. 6 C), and that the colocalization rate increased from 38.1 to 73.2% over time with the internalization of VEGFR2 (Fig. 6D-E), suggesting YULINK involved in VEGF/VEGFR2 internalization. Blocking uptake of VEGF was also observed in CTRL or *YULINK*-knockdown cells with anti-VEGF antibody treatment (Fig. 6 A), suggesting VEGF was essential for VEGF/VEGFR2 internalization. Furthermore, the binding of VEGF to cell surface increased by 1.69 folds after shRNA knockdown of *YULINK* (Fig. 6 F), suggesting more uptake of VEGF were occurred in CTRL cells. In addition, the amount of VEGFR2 in the membrane fraction was increased to 2.06 folds after similar knockdown (Fig. 6G), consistent with the observed lower internalization of VEGF/VEGFR2 in *YULINK*-knockdown HUVECs. These results indicated that VEGF/VEGFR trafficking is functionally linked to YULINK.

**Fig. 7:**
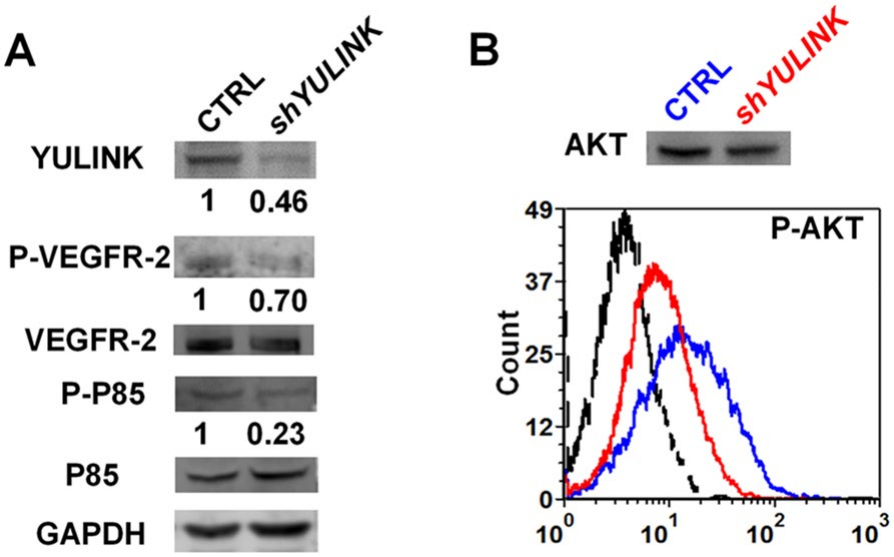
*YULINK*-knockdown dampens PI3K signaling pathway downstream of VEGFR2. **A** Western blots of total cell lysates isolated from *YULINK*-knockdown and vector control HUVECs. Phosphorylation (indicating activation) of VEGFR2 and PI3K subunit P85 was analyzed, with VEGFR2 and PI3K subunit P85 serving as the respective internal controls. GAPDH was detected as a loading control. Band intensities were quantified using the ImageQuant TL software (GE Healthcare), normalized relative to the quantity of their respective control, and expressed as percentages of the value. **B** Phosflow analysis of phosphor-AKT (Ser473) levels in *YULINK*-knockdown (red) and vector control (blue) HUVECs. An average of 3,000 gated events was collected on a Canto flow cytometer. Data were analyzed using DIVA software. The dashed profile indicates background staining with a control IgG mAb. Total AKT served as the internal control

The phosphatidylinositol-3 kinase (PI3K) signaling pathway is downstream of VEGFR2 signaling and has been implicated in the specification of vein identity. PI3K promotes venous cell fate by blocking arterial p42/44 mitogen-activated protein kinase (MAPK; extracellular signal-regulated kinase (Erk)) activation [28]. To determine whether YULINK influences PI3K signaling, we examined the activation of PI3K and downstream AKT using WB or Flow cytometry (Fig. 7 A and B). Compared to the CTRL cells, the protein level of YULINK, P-VEGFR2 and P-P85 were 46%, 70% and 23% in *YULINK*-knockdown cells, respectively (Fig. 7 A and B). These results indicated that activation of these proteins was reduced, suggesting that YULINK may be involved in VEGF/VEGFR signaling pathway.

## Discussions

In this study, we have demonstrated that knockdown of *YULINK* in zebrafish embryos resulted in the defective venous formation and abnormal vascular plexus formation. In addition, knockdown of *YULINK* in HUVECs resulted in reduction of endothelial cell migration, inhibition of tube formation, and significant decrease of phosphorylated EPHB4. It was previously reported that *de novo* formations of embryonic vessels were differentiated from vascular endothelial progenitor cells, which undergo migration and coalescence to form the primordial vascular network [2]. This primordial vascular network then sprouted ventrally and migrate to develop into the EPHB4-expressing venous cells. Subsequently, they were segregated from Ephrin B2-expressing arterial cells, leading to the formation of cardinal vein and dorsal aorta [3], separately. All these studies suggested that YULINK may involve in migration and tube formation of venous cells via EPHB4-mediated pathway.

Yeast two-hybrid experiment using Yulink showed that six proteins were identified: EPS15, RAB33B, TICAM2, ANKRD44, DENND4C, and LCA5L. The EPS15, RAB33B and TICAM2 proteins were further found to interact with YULINK using FLIM-FRET, immunoprecipitation, or various imaging techniques. Additionally, it was demonstrated that YULINK colocalizes with endosome markers (Clathrin and RHOB), which are critical for proteins assembly throughout endocytosis and regulate vasculogenesis and angiogenesis [18, 26, 29–34]. Several studies have implicated RHOB regulating endothelial cell responses to inflammatory signals but also modulating vascular function and angiogenesis [30–33], showing that YULINK may play a role in vasculogenesis through endocytosis. Previously it was reported the EPS15 plays role in Clathrin-coated vesicle formation at the plasma membrane [35, 36], the RAB33B regulates intra-Golgi retrograde trafficking pathways [21, 37], and endosomal localization of TICAM2 is a prerequisite for interferon induction [22, 38]. All these studies suggested that YULINK may involve in endocytosis through these endosome related proteins, EPS15, RAB33B or TICAM2. On the other hand, the other three interacting proteins, ANKRD44, DENND4C, and LCA5L were little known. The DENND4C may regulate complex of proteins trafficking [39] and ANKRD44 and LCA5L were known to have extremely low expression in HUVECs. We would await future studies to investigate these three proteins in details.

Our studies showed that knockdown of *YULINK* led to the loss of venous vasculature, but not dorsal aorta and arterial intersegmental vessels in the zebrafish. Then, we further examined the role of *YULINK* in HUVECs via loss of function and protein-protein interaction assays. Currently we did not know the role of *YULINK* in human artery development, waiting for future studies.

In the studies, knockdown of *YULINK* were shown to have decreased uptake of VEGF and reduced phosphorylation of VEGFR2, PI3K and Akt in HUVECs. Moreover, YULINK colocalized with VEGFR2 in endothelial cells, and the colocalization rate increased with VEGFR2 internalization over time. Previously it was reported the VEGF signaling was linked to the internalization of VEGFR2 through clathrin-dependent endocytosis [39–45]. Our studies and literature reports suggested that when VEGFR2 recognize its ligand VEGF, YULINK may further modulate internalization of VEGF/VEGFR2 complex, downstream signaling, and vascular formation.

## Conclusion

Taken together, these results showed that YULINK was required for the vasculogenesis of zebrafish and tube formation of HUVECs. FLIM-FRET, immunoprecipitation, as well as imaging technologies showed that YULINK colocalized with endosome related proteins (EPS15, RAB33B and TICAM2) and endosome markers (Clathrin and RHOB). Knockdown of YULINK decreased phosphorylated EPHB4 as well as the internalization of VEGF and VEGFR2 in HUVECs. Thus, our results support the hypothesis that YULINK participates in endocytosis processes which may involve in vasculogenesis as illustrated in Additional file 1: Fig. S6.

## Materials and methods

### Animals

Breeding and maintenance of TL strain zebrafish, as well as collection and staging of embryos, were performed according to standard procedures [46] and approved by Institutional Animal Care & Utilization Committee of the Academia Sinica. Selected embryos were reared in egg water treated with 0.003% 1-phenyl-2-thiourea to inhibit pigmentation [46]. Developmental times refer to hours (hpf) or days (dpf) post-fertilization. Transgenic *gata1:DsRed* zebrafish [47] were acquired from Dr. Leonard I. Zon at the Howard Hughes Medical Institute, and crossed to transgenic *Tg (fli1:EGFP)*^*y1*^ fish [48] acquired from Dr. Brant M. Weinstein at the NIH. The *flt1*^*enh*^:*RFP* transgenic line [49] was obtained from Dr. Stefan Schulte-Merker at the Hubrecht Institute, and crossed with transgenic *Tg (fli1:EGFP)*^*y1*^ fish to characterize arterial development in zebrafish *YULINK*-knockdown embryos.

### Cell culture

Human umbilical vein endothelial cells (HUVECs) were purchased from Lonza (Walkersville, MD, USA) and grown for 2–6 passages in EGM2 culture media (Lonza, Basel, Switzerland) supplemented with 2% fetal bovine serum (FBS). The cultures were maintained in a humidified atmosphere with 5% CO_2_ at 37 °C. HEK-293 T cells were cultured in DMEM with 10% FBS.

### Morpholino (MO) knockdown

Zebrafish embryos were obtained by natural mating, and MO microinjection was performed at the 1 ~ 4 cell stage. The *YULINK*-MO antisense oligonucleotide (5′-GGCAGGACAGTGGCTTGTTCAGTGC-3′) and its 5 bp mismatch MO negative control (5′-GGCtGcACAGTcGCTTcTTCAcTGC-3′) were used following our previous publication [13]. Embryos positioned in an agarose injection chamber were injected with 5–10 ng of MO in 4.6 nl of Danieu buffer (58 mM NaCl, 0.7 mM KCl, 0.4 mM MgSO_4_, 0.6 mM Ca(NO_3_)_2_, 5 mM HEPES, pH 7.6) using a Narishige micromanipulator and needle holder (Narishige, Tokyo, Japan). The specificity of the *YULINK*-MO was demonstrated in our previous publication [13].

### Yeast two-hybrid experiments

Yeast two-hybrid experiments were performed using a Normalized Universal Human Mate & Plate Library and the Matchmaker Gold System (Clontech, Mountainview, CA). Human *YULINK* cDNA was amplified by PCR with the following primers: 5’-CATGGAGGCCGAATTCATGAGCGGTACCAAACCTGATATTT-3’ (forward primer) and 5’-GCAGGTCGACGGATCCTTATGGCTGGACAGTCTCTGCAGGTA-3’ (reversed primer). The underlined sequences are EcoRI and BamHI restriction endonuclease sites.

The PCR construct was cloned in-frame with the *GAL4* DNA-BD of the pGBKT7 DNA-BD vector using the EcoRI and BamHI restriction sites within the multiple cloning site. The resulting *YULINK* pGBKT7 plasmid was used to transform Y2H Gold yeast cells. Appropriate positive and negative control matings, and auto-activation and toxicity experiments were performed. The Mate and Plate Library (1 mL) was combined with the bait strain (5 mL) and 2× YPDA media (45 mL, with 50 µg/mL kanamycin). Cells were incubated for 24 h at 30 °C with shaking. The cells were pelleted, washed with 50 mL of 0.5× YPDA (50 µg/mL kanamycin), and re-suspended in 10 mL of 0.9% NaCl. Next, 100 µl of 1:10, 1:100, 1:1,000; and 1:10,000 dilutions of the mated culture were spread onto 100-mm SD/-Trp, SD/-Leu, and SD/-Trp/-Leu plates. The remainder of each culture was plated individually onto 100- and 150-mm SD/-Trp/-Leu/X-αGal/Aureobasidin A plates. The plates were incubated at 30 °C for 5 d. Positive colonies were re-analyzed by streaking onto quadruple drop-out (SD/-Leu/-Trp/-His/-Ade) plates containing X-αGal/Aureobasidin A. Aliquots of blue colonies that had been serially selected were used for PCR with flanking primers specific for the pGAD-T7-RecAB plasmid, to generate insert DNA for sequencing.

### FLIM-FRET measurement

FLIM-FRET measurements were made as previously described [24, 50], with the following modifications. A Leica TCS SP5 equipped with multiphoton fluorescence lifetime imaging microscopy (Leica TCS-SP5-AOBS-MP) system (Wetzlar, Hesse, Germany) was used for confocal imaging and to measure fluorescence lifetime. A water immersion objective (Leica, 63×/0.9 APO) was employed both for focusing laser light onto the samples and for collecting fluorescence emissions from the samples. The fluorescence lifetime for each image pixel was recorded using the time-correlated single photon counting technique (SPEC-830 TCSPC modules, Becker & Hickl, Berlin, Germany). For FLIM measurements, AcGFP (green color) was excited at 440 nm using a pulsed-laser diode. Fluorescence was detected by a single photon avalanche photodiode (SPAD) with a 470 ± 15 nm band pass filter. Electrical signals were processed by the Time-Harp 200 PC card (PicoQuant, Berlin, Germany). Analysis of FLIM images was performed using the SymPhoTime software (PicoQuant), providing the instrument response function. To produce a reliable histogram of fluorescence lifetimes, we measured 15–30 cells in each experiment and recorded 50,000 photons/cell. FLIM pictures were accumulated for 90 s (60 frames with an average photon count rate of 2–4 × 10^4^ counts/s).

### Colocalization using super-resolution imaging and confocal microscope

Imaging of the samples, either HUVECs or HEK-293 T cells, was co-transfected with plasmids carrying AcGFP or DsRed fluorescence, and carried out on a Leica SR-GSD microscope. Images were taken in TIRF (total internal reflection fluorescence) mode at a rate of 100 frames per s. The setup consisted of the following components: an inverted microscope (DMI6000 B, Leica Microsystems), a 1.47-NA TIRF objective, a 488-nm fiber laser (for green fluorescence), a 532-nm fiber laser (for red fluorescence), and an EMCCD camera. The super-resolution images were taken in TIRF mode at 100 frames per s and reconstructed from a series of ~ 5,100 images, giving a total measurement time of about 1 min for each color channel. Pixel size in the image was 100 nm. Quantitative colocalization ratio analysis of multicolor fluorescence images was performed with the Imaris software suite version 7.6 (Bitplane, Zurich, Switzerland).

Each cell sample was placed under a coverslip, sealed, and examined under 100× objective magnification using a Leica TCS-SP5-MP-SMD confocal system (Leica Microsystems). Images of the two different fluorochromes were collected at 1-µm-thick optical sections using Leica software (Leica Microsystems). The colocalization analysis was done with the Imaris software suite, version 7.6.

### Pharmacological inhibitor treatment

Embryos were manually dechorionated and incubated with the inhibitor chlorpromazine (60 nM), starting from 16 hpf and continuing until the indicated developmental stage.

### Knockdown of YULINK in HUVECs 

GIPZ lentiviral shRNAmir human *YULINK* shRNA vector (Clone ID. V3LHS_374795 and gene access no. NM_019005) and its empty vector control (catalog #RHS4349) were obtained from Open Biosystems/Thermo Scientific (Huntsville, AL, USA). A small hairpin RNA (shRNA) with a tight hairpin turn was used to silence target gene expression of *YULINK* via RNA interference. To establish stable HUVECs expressing *YULINK* shRNA, primary HUVECs were infected with the recombinant retrovirus for 48 h. Infected HUVECs were selected using 2.5 µg/mL puromycin at 72 h after infection. Drug resistant cells were expanded for further studies.

### In vitro angiogenesis assays

In vitro angiogenesis assays were performed to investigate endothelial cell network formation of capillary-like tubes and migration. For the Matrigel network formation assay, each well of a 12-well Falcon tissue culture plate was evenly coated with 150 µl Matrigel (BD Biosciences, Bedford, MA, USA). At 24 h post-transfection, 6 × 10^4^ HUVECs in complete EGM-2 media were seeded per well, in triplicate. Network formation was assessed after 16 h by photographing the matrices using an Olympus IX71 inverted light microscope and a DP70 digital camera (Olympus America, Center Valley, PA, USA). Three independent fields were acquired from each well and the morphological aspects of the tube network quantified using the angiogenesis analyzer plugin [51] for ImageJ [52]. Cell migration was monitored using CIM-Plate 16 devices and the xCELLigence DP system (Roche Diagnostics, Mannheim, Germany). In this system, 1.6 × 10^4^ HUVECs (either transfected with *shYULINK*, vector control, or untreated) in normal culture media without FBS were seeded into the upper chamber. This upper chamber was then placed on the lower part of a CIM-device containing complete EGM-2 growth media as an attractant. Cell migration was monitored over a period of up to 24 h, by measuring changes in the impedance signal of the underside of the CIM-plate membrane (Roche Diagnostics).

### Whole-mount in situ hybridization

Collection and staging of embryos were performed as described above. Embryos were fixed overnight at 4 °C in 4% paraformaldehyde buffered with 1x phosphate-buffered saline (PFA/PBS). For whole-mount in situ hybridization, the following DIG-labeled RNA probes were prepared from linearized plasmids using the DIG RNA labeling kit (Roche, Basel, Switzerland): (1) an antisense probe of the ephrinB2a gene prepared from XhoI-digested plasmid (provided by Dr. Joyce Jean Lu at Genomics Research Center, Academia Sinica) using T3 RNA polymerase, and (2) a dab2 antisense probe prepared from SacII-digested pCMV-SPORT6.1-dab2 (purchased from Open Biosystems, Huntsville, AL, USA) with T7 RNA polymerase. Anti-DIG antibody conjugated to alkaline phosphatase (AP) was prepared as previously described [23, 24]. After hybridization, embryos were incubated with anti-DIG antibody conjugated to AP and developed with NBT-BCIP reagents.

### Antibodies, recombinant proteins, and phosphorylated EPHB4 ELISA

Antibodies and recombinant proteins were purchased from the following companies: α-EPS15 antibody (PAB12437), α-TICAM2 antibody (ab173389), and α-VEGF Receptor 2 from Abcam (Cambridge, UK); α-RAB33B antibody (GTX116390) and α-GAPDH from GeneTex (Irvine, CA, USA); α-Clathrin (C-8034) from Sigma-Aldrich (Saint Louis, MO, USA); α-AKT (C67E7) from Cell Signaling Technology Inc. (Danvers, MA, USA); α-Na^+^/K^+^-ATPase α1 (sc-21,712) from Santa Cruz Biotechnology Inc. (Santa Cruz, CA, USA); and α-EPHB4, biotinylated human VEGF_165_, and biotinylated Ephrin-B2 Fc from R&D Systems Inc. (Minneapolis, MN, USA). Measurement of phosphorylated EPHB4 levels in 40 µg protein extracts was performed using a sandwich ELISA kit (R&D Systems) according to the manufacturer’s instructions.

To produce specific monoclonal antibody against human YULINK, the full-length *YULINK* cDNA-expression plasmid was transfected into CHO cells by Lipofectamine 2000 reagent (*Thermo Fisher* Scientific, Waltham, *MA, USA*). The YULINK-expressing CHO cells, in which expression was confirmed by Western blot, were obtained by G418 selection. Then the monoclonal antibody against YULINK was prepared with a hybridoma technique provided by Abnova (Taipei, Taiwan).

### Ligand internalization assay

To analyze the internalization of VEGF in vitro, HUVECs (*YULINK*-knockdown and negative controls) were incubated in serum-free growth media containing 30 ng/mL VEGF-biotin or anti-VEGF blocking antibody pretreated with VEGF-biotin at 37 °C for the indicated period of time, before being fixed with 4% formaldehyde in PBS for 15 min. For the specificity of VEGF internalization, anti-VEGF-blocking antibody (R&D Systems) was pre-incubated with VEGF-biotin for 15 min at room temperature. After three washes with PBS of 5 min each, cells were permeabilized for 15 min with 0.5% Triton-X 100 in PBS. Alexa-Fluor-594-conjugated streptavidin (1:500, Thermo Fisher Scientific, Pittsburgh, PA) was incubated with cells for 1 h at room temperature to visualize internalized ligands. Quantification was performed with MetaMorph software (Molecular Devices).

### Immunoprecipitation (IP)

Cells were harvested in PBS and lysed for 30 min on ice in IP lysis buffer (10 mM Tris/Cl pH 7.5, 150 mM NaCl, 0.5 mM EDTA, 0.5% NP-40) freshly supplemented with 1 mM PMSF and EDTA-free protease inhibitor cocktail. Target proteins were captured directly onto GFP-Trap agarose beads (ChromoTek, Hauppauge, NY, USA) for AcGFP-tagged YULINK protein. Complexes were washed 3 times with wash buffer (10 mM Tris/Cl pH 7.5, 150 mM NaCl, 0.5 mM EDTA) supplemented with protease and phosphatase inhibitors. IP from cells that do not express GFP-tagged protein were used as negative controls.

### Mass spectrometry (MS) analysis

Affinity purified YULINK was processed by in-gel digestion. AcGFP-YULINK protein was purified by using GFP-trap beads following the IP protocol described above, with three additional washes with wash buffer. A portion of the purified YULINK was separated on SDS-PAGE and peptides were recovered using the in-gel method. Peptides from in-gel samples were loaded onto C18 spin columns (Thermo Fisher Scientific, Rockford, lL, USA). Peptides were eluted from columns with 70% acetonitrile and separated on a C18 column using an online nano-LC (Proxeon, Odense, Denmark) coupled to an LTQ-orbitrap velos mass spectrometer (Thermo Fisher Scientific, Bremen, Germany). In each full scan the 10 most abundant peptides were selected for higher-energy C-trap dissociation (HCD) fragmentation. Raw data files were processed and analyzed using Mascot Daemon software (Matrix Science, Boston, USA).

### Statistical methods

Statistical analyses were conducted using SigmaPlot software (Systat Software, Hounslow, London, UK). A Student’s t-test was conducted for two-sample analyses. Two-way ANOVA was performed to examine the impacts of treatment, time, and “treatment x time interaction” on the VEGF uptake of *YULINK*-knockdown cells.

## Supplementary Information

**Additionl file 1:** **Fig. S1.** YULNK-knockdown does not affect formation of arteries. **Fig. S2.** Silencing of YULINK inhibits capillary tube formation of endothelial cells. **Fig. S3.** Co-immunoprecipitation of YULINK with its interacting proteins. **Fig. S4.** Confocal imaging analysis of colocalization of YULINK with its interacting proteins and an endosome marker in HUVECs.** Fig. S5.** Over-expression of endosome related proteins rescued the phenotype of YULINK-knockdown HUVECs. **Fig. S6.** A model illustrates the involvement of YULINK in venous-fated angioblast. **Table S1.** Proteins identified in yeast two-hybrid experiments that interact with YULINK.

**Additional file 2: Video S1.** Knockdown of *YULINK* led to defective venous formation in zebrafish.

## Data Availability

The datasets used and/or analyzed during the current study are available from the corresponding author on reasonable request.

## Abbreviations

aISV: Arterial intersegmental vessels
CA: Caudal artery
CV: Caudal vein
DA: Dorsal aorta
FLIM-FRET: Förster resonance energy transfer by fluorescence lifetime imaging microscopy
HUVECs: Human umbilical vein endothelial cells
IP: Immunoprecipitation
KD: Knockdown
MO: Morpholino
MS: Mass spectrometry
PCV: Posterior cardinal vein
vISV: Venous intersegmental vessels
