# Supplementary material for: YULINK regulates vascular formation in zebrafish and HUVECs
Source: Biol Res. 2023 Feb 27;56:7. doi: 10.1186/s40659-023-00415-8 (PMC9969694; doi:10.1186/s40659-023-00415-8)
Supplement: Supplementary file 1 — Additionl file 1: Fig. S1. YULNK-knockdown does not affect formation of arteries. Fig. S2. Silencing of YULINK inhibits capillary tube formation of endothelial cells. Fig. S3. Co-immunoprecipitation of YULINK with its interacting proteins. Fig. S4. Confocal imaging analysis of colocalization of YULINK with its interacting proteins and an endosome marker in HUVECs. Fig. S5. Over-expression of endosome related proteins rescued the phenotype of YULINK-knockdown HUVECs. Fig. S6. A model illustrates the involvement of YULINK in venous-fated angioblast. Table S1. Proteins identified in yeast two-hybrid experiments that interact with YULINK. [file 40659_2023_415_MOESM1_ESM.docx]

**SUPPLEMENTARY INFORMATION**

**Results:**

**Fig. S1. *YULNK*-knockdown does not affect formation of arteries.**

A: Whole-mount *in situ* hybridization in wild type (WT) and *YULINK*-knockdown (MO) at 2 dpf embryos. Embryos were hybridized with DIG-labeled RNA probes for the artery marker *efnb2a* (upper panels) and the vein marker *dab2* (lower panels). Note the dramatic reduction in expression of the vein marker *dab2* in the PCV (indicated by a bracketed line) in the *YULINK*-knockdown embryo at 2 dpf. B: Knockdown of *YULINK* displayed normal artery formation at 2 dpf. Confocal images of vasculature are shown. The double Tg *(fli1:EGFP; flt1:RFP)* embryos displayed green fluorescent protein EGFP and red fluorescent protein DsRed and in all endothelial cells and arteries, respectively. The left panel shows *Tg* embryos (WT) with normal formation of arteries and veins. The right panel shows *YULINK*-knockdown morphant (MO) with normal arteries and disappeared most vISVs. WT, wild type; MO, *YULINK*-knockdown morphants; DA, dorsal aorta; PCV, posterior cardinal vein; CA, caudal artery; CV, caudal vein; aISV, arterial intersegmental vessel; vISV, venous intersegmental vessel. Scale bars indicate 100 μm.


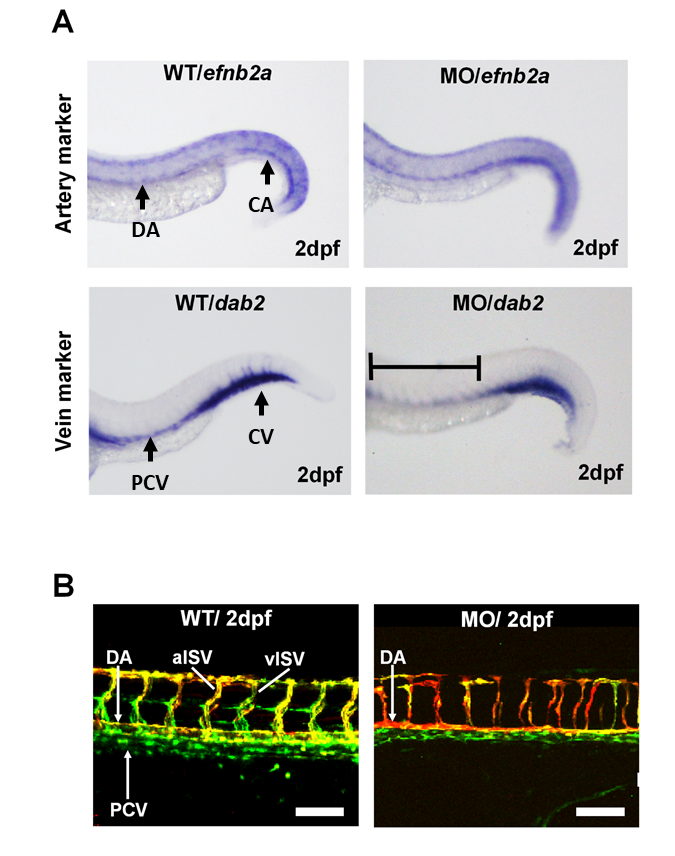


**Fig. S2. Silencing of YULINK inhibits capillary tube formation of endothelial cells.**

A: Knockdown efficiency of shRNA targeting *YULINK* in HUVECs by qPCR. (mean ± SD, *n* = 4, ***p* < 0.001). B-F: HUVECs were transfected with GFP, vector-control (CTRL), or *YULINK* shRNA (*shYULINK*) plasmids, and then endothelial cell network formation was assessed using a Matrigel assay. After 8 h of incubation at 37 °C, the formation of tubes was examined by phase-contrast microscopy. Microscopic images were analyzed using NIH ImageJ software with Angiogenesis Analyzer plugin, and segments are shown in magenta, branches in green, and master junctions in red. B: Representative phase contrast micrographs are photographed at 40x magnification. C: Examples of ImageJ processed images for quantification of tube networks and nodes, D: Total length, E: Number of tube junctions, F: Total branching length, and Number of tube nodes were quantified in panel C. WT, GFP, and CTRL cell clones were capable to form tubular network. After *YULINK* was knockdown, cells did not form extensive network. Results represent mean ± SD of 2 replicate experiments with 3 measurements per treatment, *n* = 6, **p* < 0.05, ***p* < 0.001.


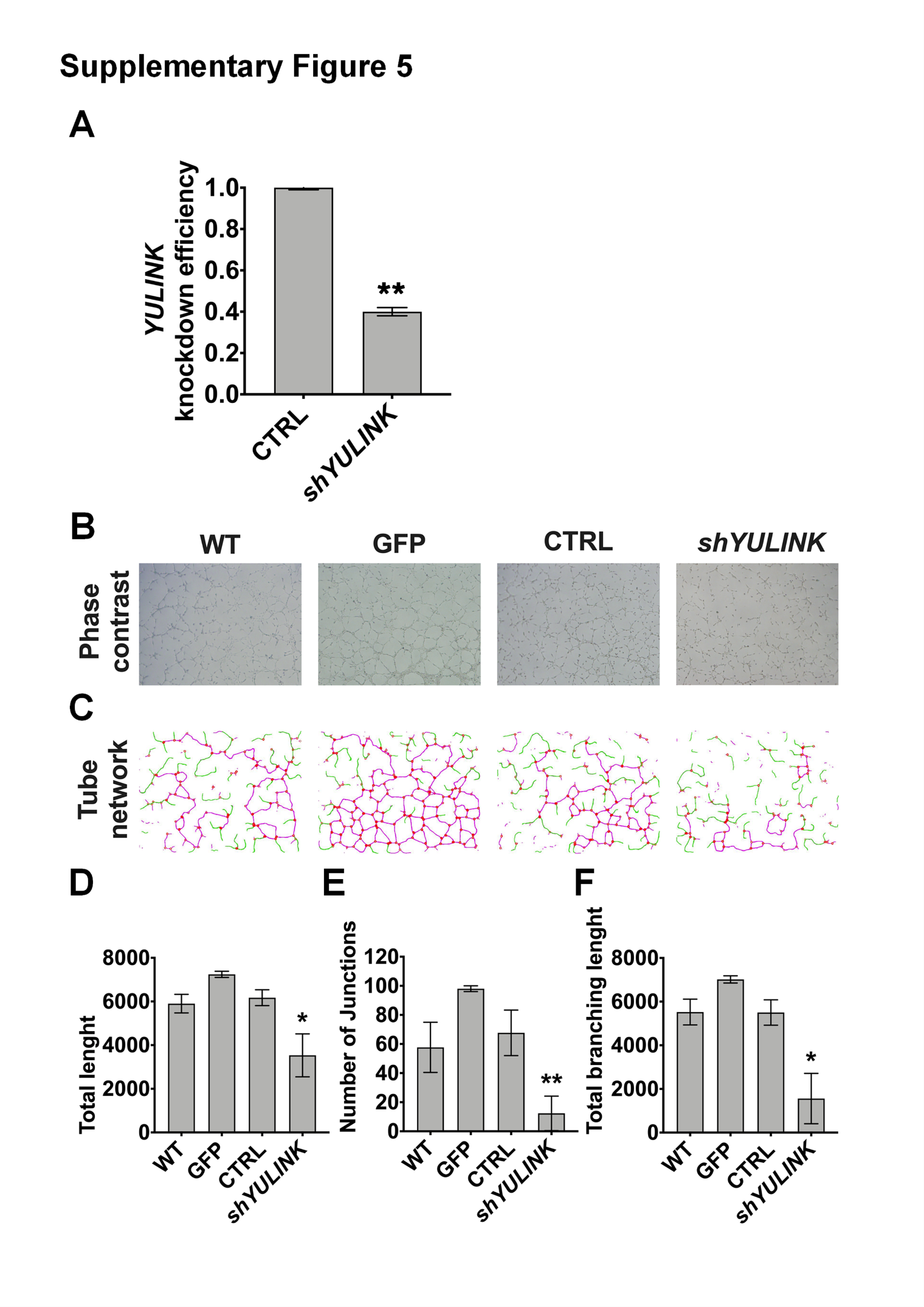


**Fig. S3. Co-immunoprecipitation of YULINK with its interacting proteins.**

Lysates from HUVECs transfected with AcGFP-YULINK and CTRL vector were subjected to immunoprecipitation with anti-GFP antibody and analyzed by immunoblotting with anti-GFP, anti-YULINK, anti-EPS15, anti-RAB33B antibody, or anti-TICAM2. IB, immunoblotting; IP, immunoprecipitation.


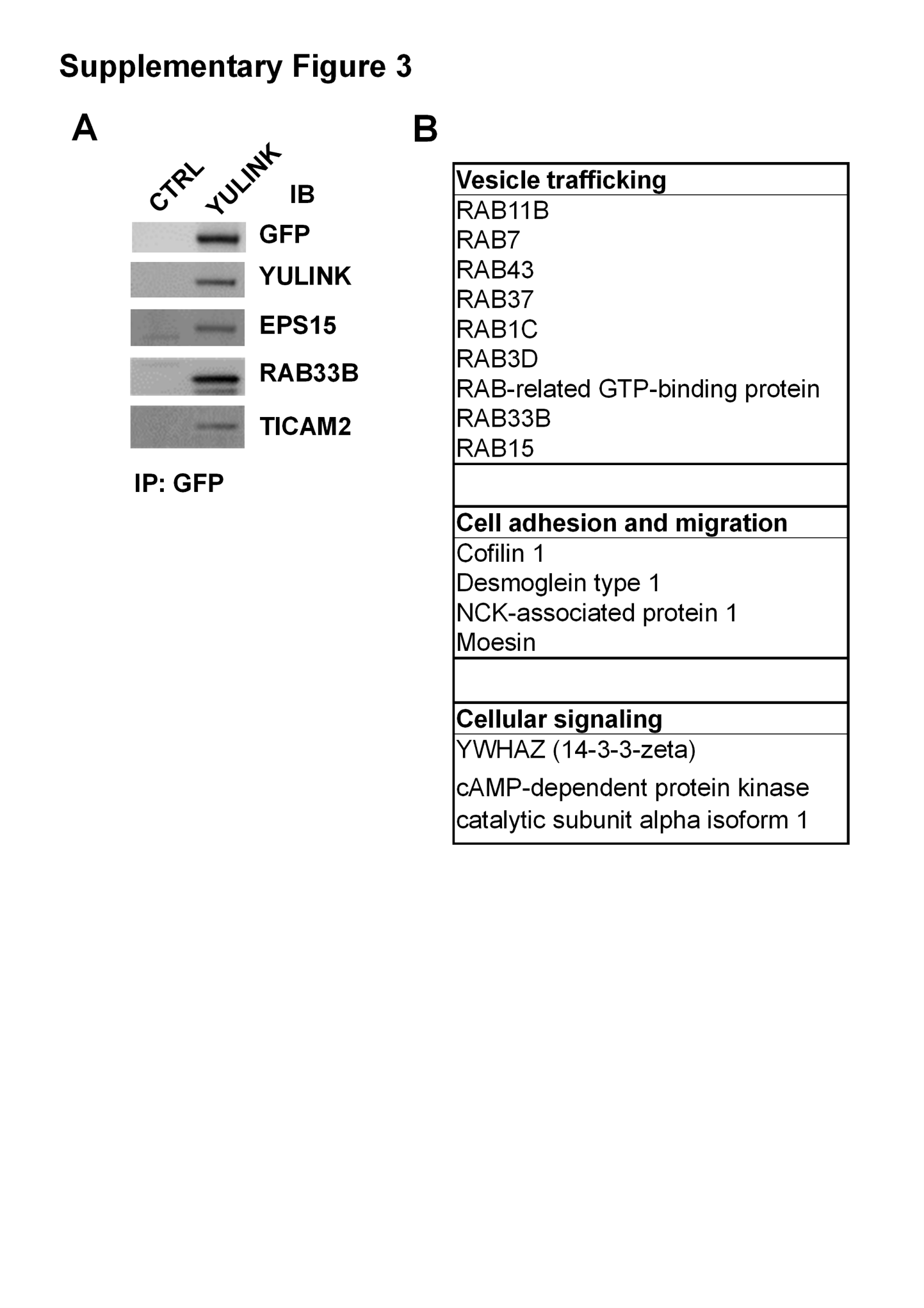


**Fig. S4. Confocal imaging analysis of colocalization of YULINK with its interacting proteins and an endosome marker in HUVECs.**

Confocal imaging analysis was used to determine whether the identified proteins colocalized with YULINK. The HUVECs were fixed, permeabilized, stained with antibodies against the proteins of interest, and analyzed by confocal microscopy. Merge images demonstrate colocalization of green- and red-stained molecules by a shift towards yellow. The colocalization percentage and Pearson correlation coefficient (r) for pixel intensity correlation between the red and green channels are displayed in far-right panel of each row. A: HUVECs were stained for YULINK (green) or for EPS15, RAB33B, TICAM2 or Clathrin (red). These colocalization ratio (with Pearson correlation coefficient r) were about 43.2% (r = 0.52), 89.6% (r = 0.85), 58.9% (r = 0.75) and 71.3% (r = 0.83), respectively. B: Clathrin is colocalized with YULINK-interacting proteins in HUVECs. HUVECs were stained for Clathrin (green) or for EPS15, RAB33B, or TICAM2 (red). These colocalization ratio (with Pearson correlation coefficient r) were about 86.6% (r = 0.86), 95.7% (r = 0.92) and 87.3% (r = 0.82), respectively.


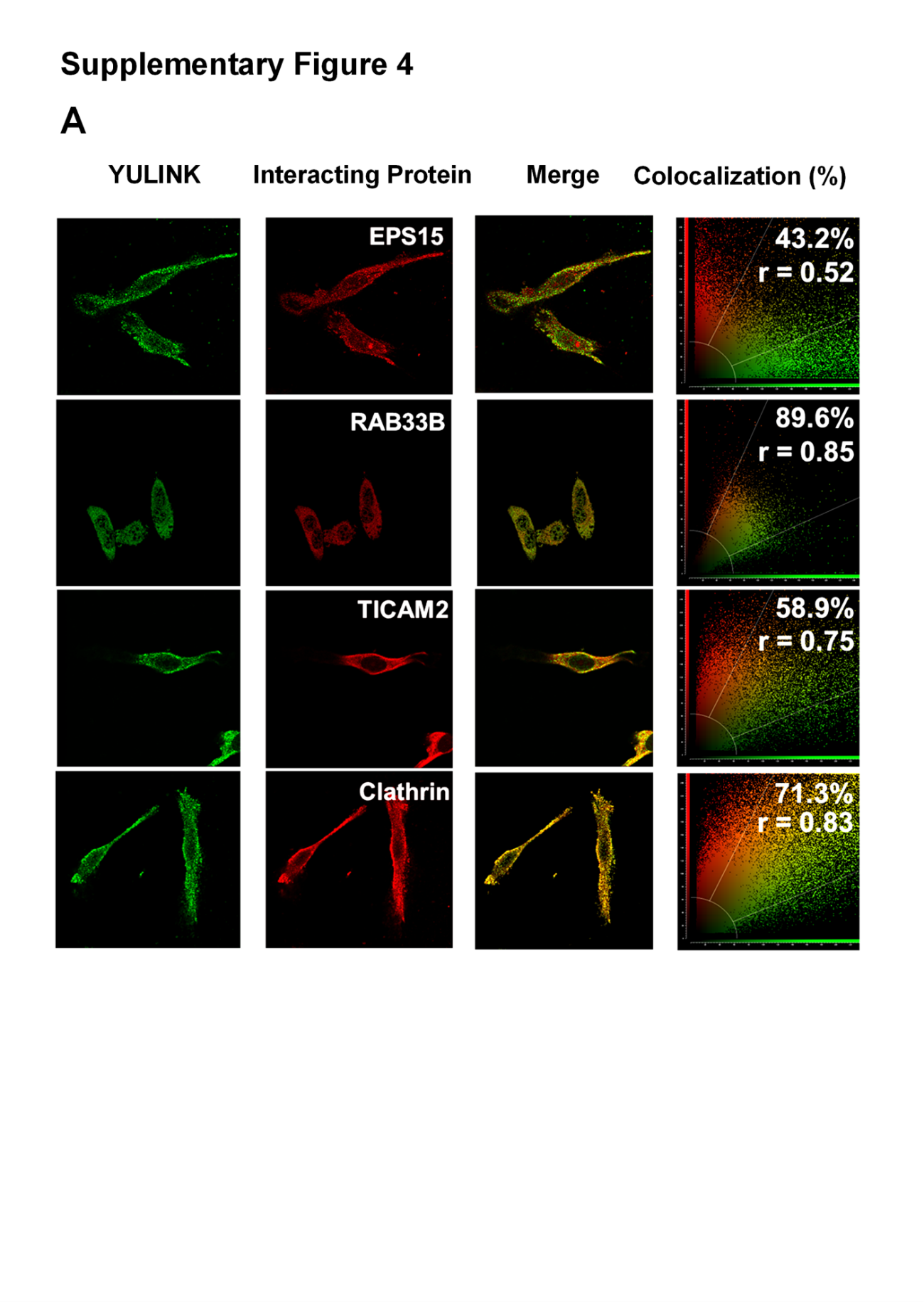


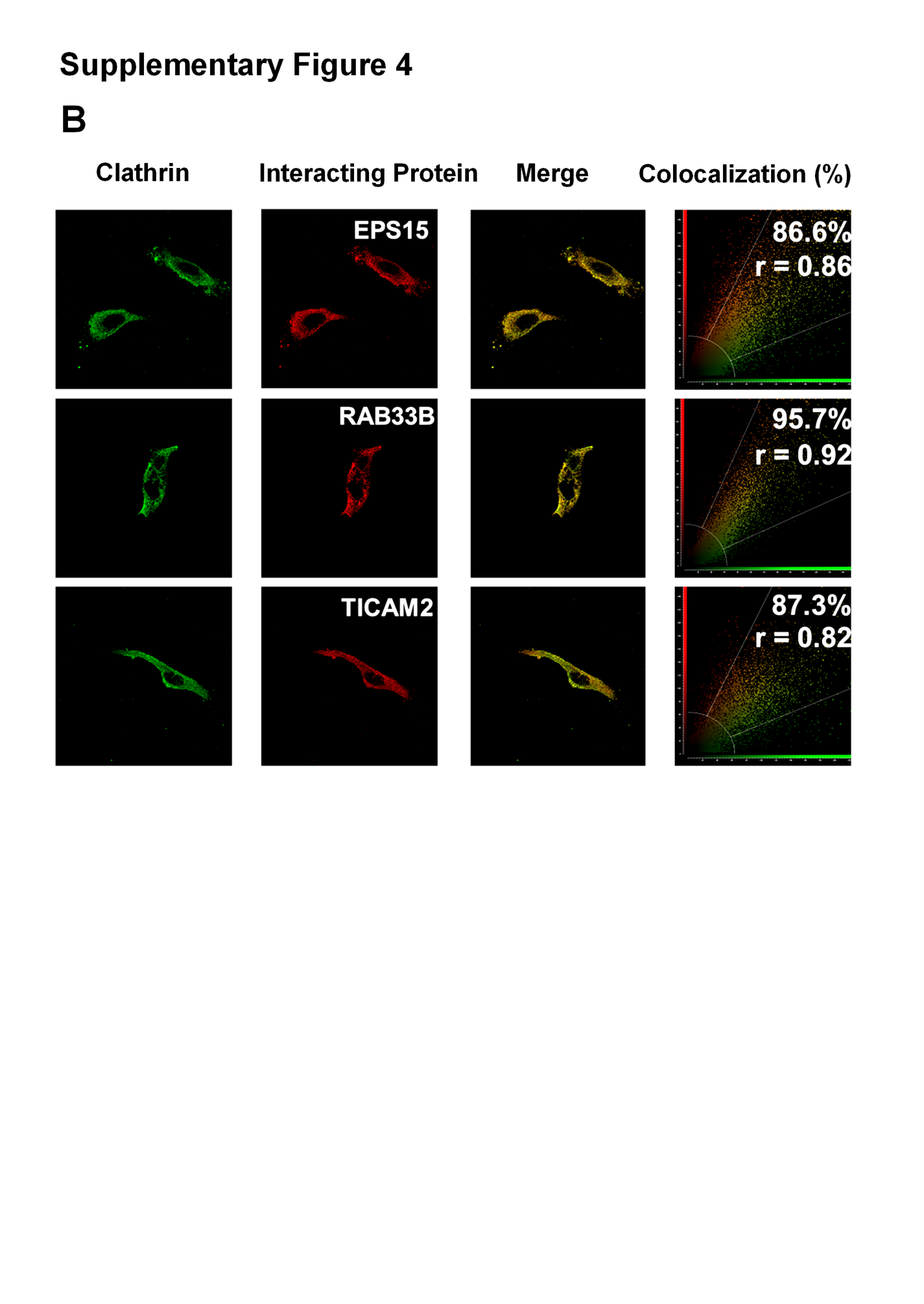


**Fig. S5. Over-expression of endosome related proteins rescued the phenotype of *YULINK*-knockdown HUVECs.**

Real-time cell migration of the transfected cells was measured using CIM plates in the xCELLigence DP system, which detects the impedance across a cell-permeable membrane. A, over-expression of DsRed-EPS15. B, over-expression of DsRed-RAB33B. C, over-expression of DsRed-TICAM2. All cells were seeded (1.5 x 10^4^ cells/well) and allowed to migrate for 20 h. Cell migration activity, expressed as cell index (*n* = 4). The quantitative comparison of the migration assayed at 9 h was expressed as cell index (indicated as red line). The p-value was less than 0.01 (**) between shYULINK and shYULINK + EPS15, and p-value was less than 0.05 (*) between shYULINK and shYULINK + RAB33B. They did not show statistically significant difference in migration between shYULINK and shYULINK + TICAM2.

**
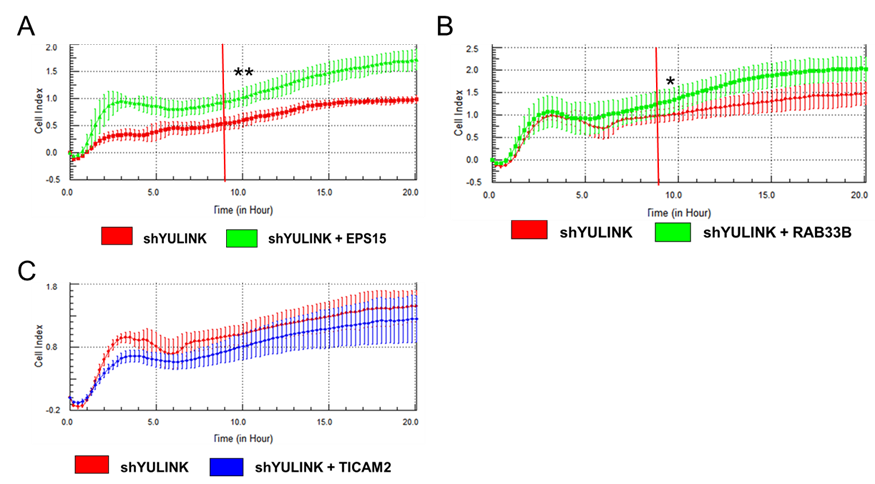
**

**Figure S6. A model illustrates the involvement of YULINK in venous-fated angioblast.**

During normal venous-fated angioblast development,

YULINK facilitates the internalization of ligand-bound VEGFR2 to endosomal compartments and induces downstream signalings, like PI3K and AKT. In *YULINK*-defective cells, the internalization of VEGFR2 and their downstream signalings were blocked. The endocytosis related protein (EPS15, RAB33B, TACAM2 or RHOB) may participate in the VEGF signaling via YULINK during venous development.

**
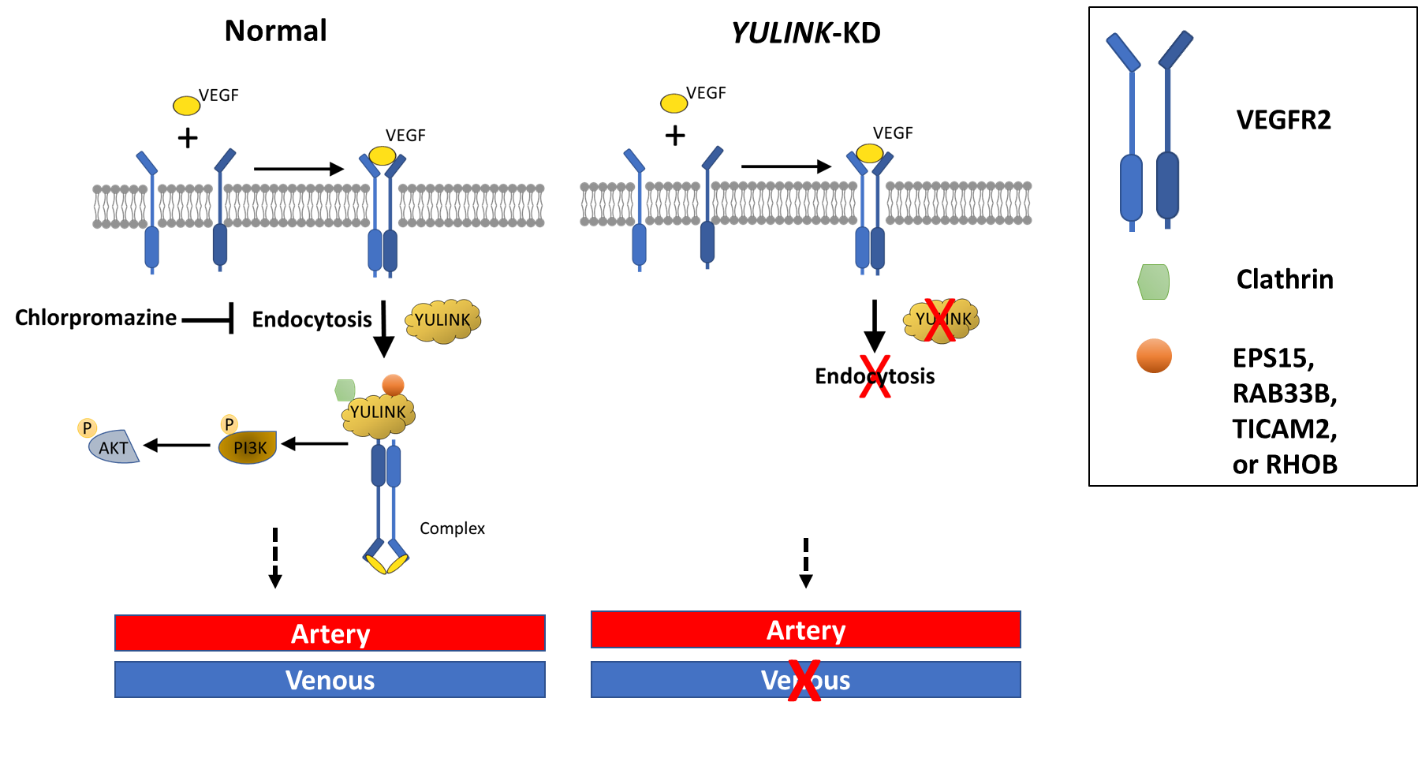
**

**Table S1. Proteins identified in yeast two-hybrid experiments that interact with YULINK.**

| Protein | Gene ID^‡^ | Accession number^£^ | Functions |
| --- | --- | --- | --- |
| ANKRD44 | 91526 | NP_710181.1 | Putative regulatory subunit of protein phosphatase 6 (PP6) that may be involved in the recognition of phosphoprotein substrates^¶^. |
| DENND4C | 55667 | NP_060395.4 | A guanine nucleotide exchange factor for the endosomal GTPase Rab10. It is potentially an important new regulator of retromer-mediated trafficking^1–3^. |
| EPS15 | 2060 | NP_001972.1  NP_001153441.1 | Present at clathrin-coated pits, and is involved in receptor-mediated endocytosis of EGF^§^. |
| LCA5L | 150082 | NP_689718.1 | Unknown. |
| RAB33B | 83452 | NP_112586.1 | Plays important roles at defined steps of vesicular transport in protein secretion and the endocytosis pathway^§^. |
| TICAM2 | 353376 | NP_067681.1 | Functions in LPS-TLR4 signaling to regulate the MYD88-independent pathway during the innate immune response to LPS. Also involved in IL1-triggered NF-kappa-B activation, functioning upstream of IRAK1, IRAK2, TRAF6, and IKBKB^¶^. |

‡Annotations described in the Entrez Gene database at NCBI.

£Accession number used in the RefSeq database.

§Entrez Gene summary

¶UniProtKB/Swiss-Prot Gene summary
